# Zn Complexes with the 4-Acyl-Pyrazol-5-One Basis and Their Thin Films

**DOI:** 10.3390/ijms27177560

**Published:** 2026-08-24

**Authors:** Alexey Gusev, Elena Braga, Alexandra Pismennaia, Valery Vlasenko, Miki Hasegawa, Mikhail Kiskin, Wolfgang Linert

**Affiliations:** 1 General Chemistry Department, Crimean Federal University V.I. Vernadsky, 295007 Simferopol, Russia; galex0330@gmail.com (A.G.); braga.yelena@ya.ru (E.B.); alex.stivenson@bk.ru (A.P.); 2 Institute of Physics, Southern Federal University, 344090 Rostov-on-Don, Russia; vgvlasenko@sfedu.ru; 3 Department of Chemistry and Biological Science, College of Science and Engineering, Aoyama Gakuin University, 5-10-1 Fuchinobe, Chuoku, Sagamihara 252-5258, Kanagawa, Japan; hasemiki@chem.aoyama.ac.jp; 4 N.S. Kurnakov Institute of General and Inorganic Chemistry, Russian Academy of Sciences, 119991 Moscow, Russia; mkiskin@igic.ras.ru; 5 Institute of Applied Physics, Vienna University of Technology, Wiedner Hauptstraße 8-10, 1040 Vienna, Austria

**Keywords:** Zn complex, pyrazolone, luminescence, thin-films, DFT

## Abstract

Three zinc(II) complexes based on 1-phenyl-3-methyl-4-acyl-5-pyrazolone were synthesized and characterized by elemental analysis, by X-ray crystallography and spectroscopy (UV-vis, fluorescence and IR). Crystallographic studies reveal that the complexes have a mononuclear structure in the solid state; however, intermolecular interactions combine the complexes into a 1D polymer chain. The complexes exhibit weak luminescence in the polycrystalline state and moderate emission in solutions and thin amorphous films. Thin films of the studied compounds were deposited on a glass substrate using thermal vacuum spraying, spin coating, and Langmuir-Blodgett technology and were studied using atomic force microscopy (AFM), X-ray spectroscopy, and fluorescence spectroscopy.

## 1. Introduction

The great progress in the chemistry of coordination compounds of transition metals with organic molecules over recent decades is associated with a wide range of functional capabilities that are not achievable with “pure” inorganic and organic compounds. Inorganic metal cations determine optical, electrical, magnetic, conductive, and catalytic properties, while the rich structural chemistry of organic molecules allows for fine-tuning target properties and provides flexibility to materials [1,2,3,4]. Among numerous applications of functional coordination compounds, the greatest successes today have been achieved through the use of photoactive complexes for creating active laser media, sensors for various toxic and explosive substances, as well as electromagnetic radiation emitters in electroluminescent devices [4,5,6,7,8].

Nevertheless, it should be noted that despite the broad library of photoactive complexes demonstrating excellent optical characteristics, their application in industrial production is limited by issues in applied materials science. Firstly, for widespread use, photoactive coordination compounds must be low-cost and based on commonly found metals in the Earth’s crust [9]. Until recently, the best optical properties were observed in complexes based on rare-earth and expensive metals (Ir, Pt, Os, Tb, Eu) [10,11,12,13,14,15,16], and only in the last decade have highly efficient phosphors been developed based on more accessible 3d-metals (Cu, Mn, Zn) [17,18,19,20,21,22,23,24]. Secondly, a lot of coordination compounds exhibit promising photo-physical characteristics in the solid polycrystalline state, but their use in devices often depends on the preservation of photoactivity in thin films as well as structural, morphological, and surface properties of thin films that significantly influence on their luminescent performance. However, only a very limited number of coordination compounds’ film-forming properties have been thoroughly studied, and consequently, their photophysical characteristics in this state are poorly studied [25,26,27].

One of the factors reducing the possibility of the formation of thin films is the low solubility and/or volatility of photoactive coordination compounds due to their polymeric nature, ionic structure, and high molecular weight. Meanwhile, the need to use complexes in thin films will also be determined by new opportunities for their application, in particular, to play a crucial role in controlling light and enabling cutting-edge technologies such as OLED and infrared optics, spintronic devices, as well as for creating lithographic matrices in microelectronics [28,29,30,31,32].

The literature describes a considerable number of technological solutions for thin films producing. However, the most applicable methods for coordination compounds are Thermal Vapor Deposition (TVD), the spin-coating method (SCM), and Langmuir–Blodgett techniques (LBT). For each method, the coordination compounds must meet specific requirements: for vacuum deposition, complexes should be thermally stable and volatile; for spin-coating—soluble in low-polarity solvents; and for LBT—possess amphiphilic properties. Clearly, the combination of all three conditions in a single compound is unlikely, which prevents direct comparison of the efficiency of different film-forming methods and the properties of the films obtained by various techniques [33,34,35,36,37].

4-Acyl-5-pyrazolones are a convenient class of organic ligands for tuning the properties of coordination compounds due to their broad chemical modification capabilities through the introduction of various substituents, while maintaining the coordination polyhedron structure [38,39,40,41]. Luminescent properties of lanthanide coordination compounds (primarily terbium and europium) with acylpyrazolones represent a quite common class of emitters in thin-film OLEDs [42,43]. Variations in substituents on the organic ligand have enabled different methods for preparing thin films of 4f-metal complexes and investigating their properties. The literature also describes zinc complexes with acylpyrazolones, exhibiting promising photoluminescent and antitumor activities [39,40,41]. However, the use of zinc complexes with this class of organic ligands for thin film fabrication remains limited and not systematically explored [44].

**Scheme 1:**
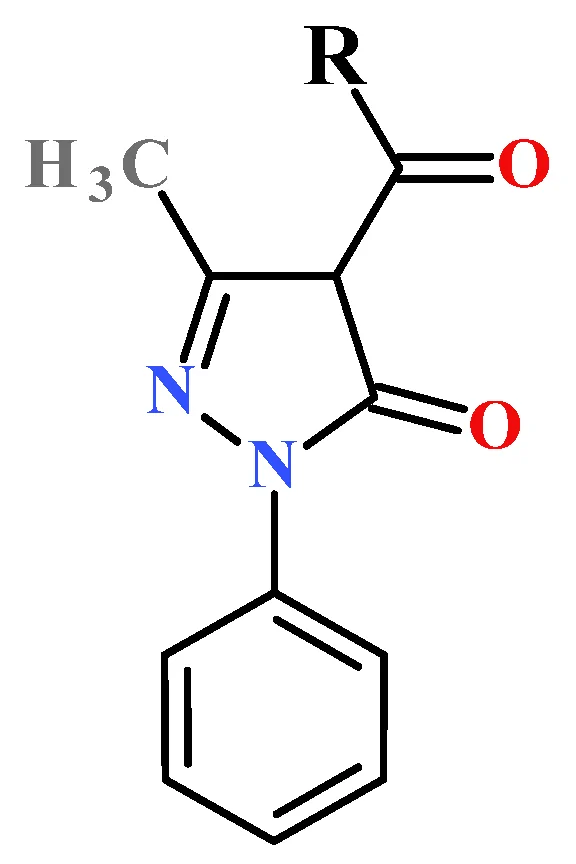
Structure of 1-phenyl-3-methyl-4-acyl-5-pyrazolone ligands (R = C_6_H_13_ (HL^1^), R = C_9_H_19_ (HL^2^), R = C_15_H_31_ (HL^3^)).

In this paper, we present the results of using zinc coordination compounds with 4-acylpyrazolones for creating thin films by various methods, and evaluating their quality and photoluminescent properties. We synthesized three coordination compounds with the general formula [Zn(L)_2_(H_2_O)], where HL^1^ = 1-phenyl-3-methyl-4-heptanoyl-5-pyrazolone (**1**), HL^2^ = 1-phenyl-3-methyl-4-decanoyl-5-pyrazolone (**2**), HL^3^ = 1-phenyl-3-methyl-4-palmitoyl-5-pyrazolone (**3**) (Scheme 1). The choice of derivatives was determined by the possibility of varying the methods of film formation and influencing their quality by changing the length of the aliphatic substituent. Based on these, thin films were produced using TVD, SCM, and LBT methods. Their photo-physical properties were characterized.

## 2. Results

### 2.1. General Characterization

**Scheme 2:**
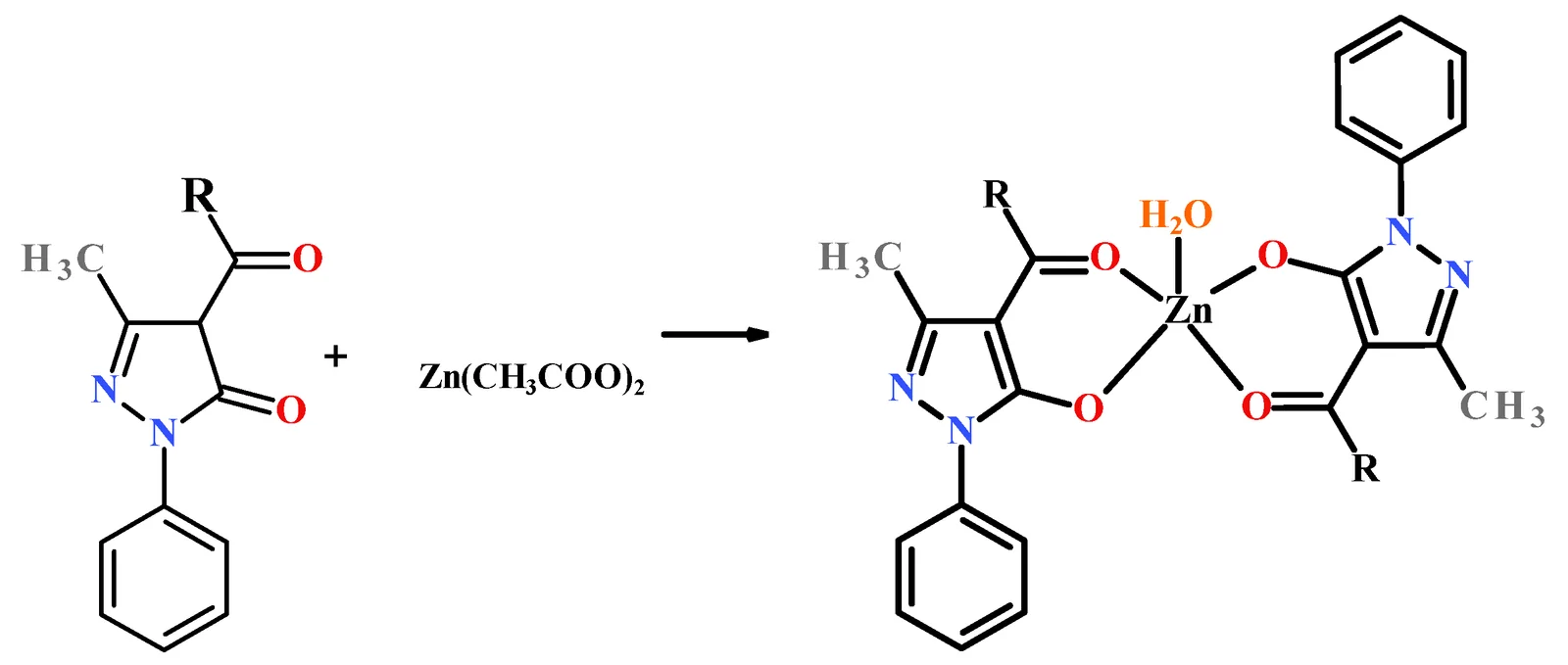
Synthetic route of zinc(II) compounds **1**–**3** (R = C_6_H_13_ (**1**), R = C_9_H_19_ (**2**), R = C_15_H_31_ (**3**)).

Complexes **1**–**3** was prepared were obtained by reacting the corresponding HL^1−3^ with zinc(II) acetate in a molar ratio of 2:1 under refluxing in methanol (Scheme 2). Crude complexes were twice recrystallized from methanol to achieve high purity of the samples. All the complexes were stable to air and moisture without any kind of decomposition even after several months. The complexes are insoluble in water, but soluble in alcohols, DMF and DMSO. Three title Zn(II) complexes were characterized by elemental analyses, FT-IR, UV–Vis, luminescent spectroscopy, and single-crystal X-ray diffraction analysis. Phase purity were confirmed by the PXRD (Figures S1–S3). The elemental analysis was consistent with their chemical formulas.

The complexation is accompanied by the deprotonation of the ligands with the transition to the enol form (Scheme 1). IR spectra of compounds **1**–**3** were compared with those of HL^1^-HL^3^ (Figures S4–S6). The most informative bands were selected for further analysis confirming the coordination mode and tautomeric form of the coordinated ligand. The IR bands for the free ligands HL^1−3^ at about 1632–1648 cm^−1^ assigned to (C=O) of the lateral chain are found to be considerably shifted to a lower frequency at about 1611–1624 cm^−1^. Moreover, in the IR-spectra of **1**–**3**, a band appears 1352–1373 cm^−1^ that correspond to the stretching vibrations of the deprotonated enol group from the pyrazolone moiety of the studied ligands. The pyrazole ring C=N stretching frequency remained at 1498–1503 cm^−1^, suggesting that the ring nitrogen does not take part in coordination. A broad band at about 3320–3350 cm^−1^ in the IR-spectra of the complexes was assigned to coordinated water.

**Figure 1:**
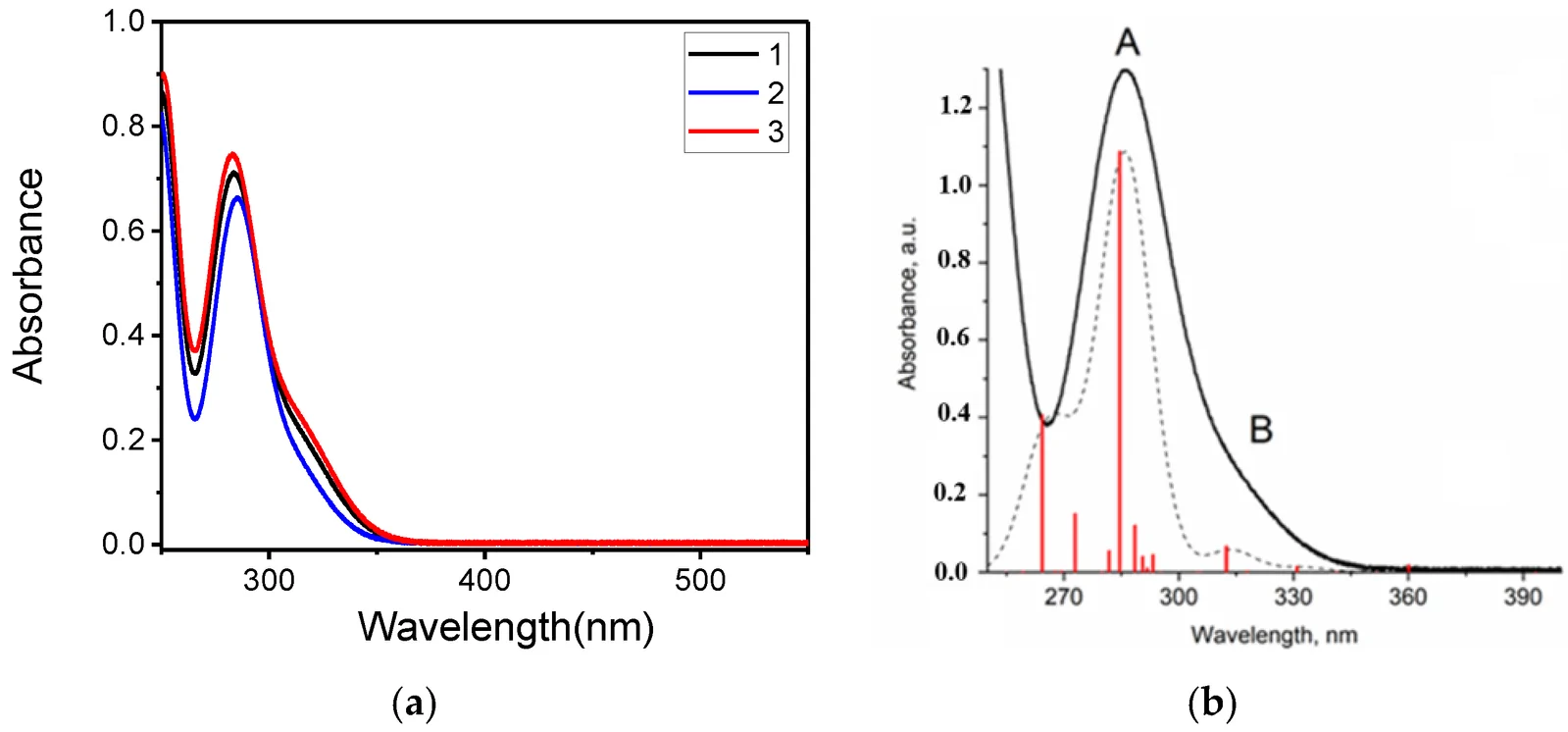
UV–Vis spectra of **1**–**3** (**a**); Experimental (solid black line) and theoretical (dashed black line) UV–Vis spectra of **3** (**b**). Individual calculated transitions are indicated by red bars, the heights of which represent the oscillator strengths. Label A means main absorption maxima while label B means long-wavelength absorption band.

UV–Vis absorption spectra of the title complexes in methanol solution were studied in the wavelength range of 250–550 nm. A strong absorption band with maxima at 282–284 nm and a shoulder in the long-wavelength region at 318–321 nm are found in the spectra of **1**–**3**. To gain deeper insight into the nature of the electronic transitions underlying the photophysical properties of the zinc complexes, time-dependent density functional theory (TD-DFT) calculations were performed for complex **3**. Figure 1 shows the experimental UV–Vis spectra of zinc complex **3**, compared with the theoretical spectra obtained from TD-DFT calculations, demonstrating good agreement between them.

**Figure 2:**
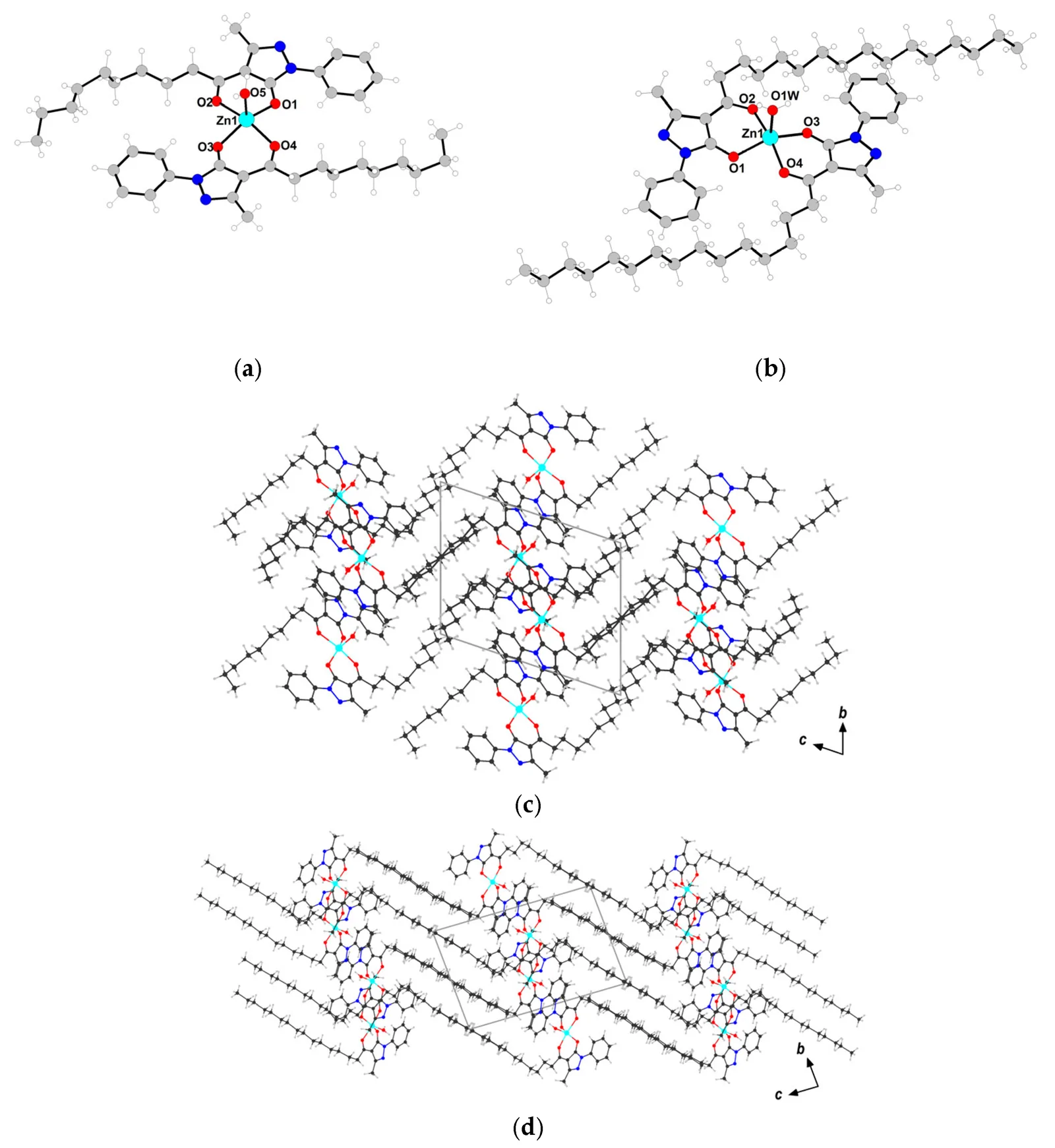
Molecular structure and crystal packing of **2** (**a**,**c**) and **3** (**b**,**d**).

According to the TD-DFT results, the long-wavelength absorption band (B) for both zinc complex is characterized not by a single dominant HOMO → LUMO transition but by a superposition of several electronic configurations. The low-energy excited state S_3_ (3.50 eV, λ = 354 nm) is primarily composed of a combination of HOMO → LUMO + 1 (~23%) and HOMO–1 → LUMO (~16%) transitions. The higher-energy excited states, which constitute the main absorption maxima (e.g., band A) in the UV–Vis spectra, arise from numerous electronic transitions from deeper-lying occupied orbitals to low-lying virtual orbitals. For instance, the intense absorption band A in the spectrum of **3** is attributed primarily to the S_29_ state (4.52 eV, λ = 274 nm, f = 0.60), with major contributions from the HOMO–4 → LUMO + 3 (8%) and HOMO–4 → LUMO (6%) transitions. Based on the DFT-calculated isosurfaces of the selected frontier molecular orbitals (FMOs) for complex **3** (Figure 2), the electron density is predominantly localized on the pyrazole fragments of the ligands for all key orbitals. The zinc atoms exhibit only a minor contribution, consistent with their filled 3d^10^ shell. The hydrocarbon moieties of the ligands also contribute insignificantly to the FMOs. Consequently, the primary character of the electronic transitions responsible for the UV–Vis spectra of **3** is assigned to ligand-to-ligand charge transfer (LLCT) between the pyrazole fragments, specifically π(L_Pyr_) → π*(L_Pyr_).

The thermal stability of the complexes is one of the key parameters determining the possibility of obtaining thin films based on them by vacuum deposition techniques and for subsequent applications [35]. The thermal stability of compounds **1**–**3** was studied by thermogravimetric analysis (Figures S7–S9). The thermal behavior is characterized by the following processes. In the temperature ranges of 77–115 °C, 82–110 °C, and 85–152 °C, mass losses of 2.5%, 2.4%, and 1.8% are observed for complexes **1**–**3**, respectively, corresponding to the removal of coordinated water molecules. The dehydration process is accompanied by an endothermic effect with a minimum at temperatures of 71 °C (complex **1**), 85 °C, and 102 °C (complex **2**). For complex **3**, the dehydration process overlaps with melting at a temperature of 82 °C.

The synthesized complexes **1**–**3** in the crystalline state exhibit very weak emission under UV excitation, with a maximum at 465–473 nm and a quantum yield of less than 1%. Conversely, solutions in acetonitrile demonstrate intense photoluminescence upon excitation at 330 nm. The emission maxima are observed at 418 (**1**), 420 (**2**), and 415 (**3**) nm. The luminescence efficiency in solutions is significantly higher than in the crystalline state and amounts to 12.8%, 11.4%, and 15.2%, respectively. The excited-state lifetimes are in the range of 3.8–4.1 ns, indicating a fluorescent emission mechanism.

### 2.2. Single-Crystal X-Ray Diffraction Analysis

According to the results of single crystal X-ray diffraction experiments, the structures of **2** and **3** are new, while the structure of **1** was determined by Li-Ying Xu and co-authors [34]. Complexes **2** and **3** crystallize in the triclinic $P\bar{1}$ space group (Table S1, Figure 2a,b). In both cases, the Zn(II) atoms are pentacoordinated by two pairs of oxygen atoms from two deprotonated acylpyrazolone ligands and one oxygen atom from a coordinated water molecule (Table S2) to form a distorted square pyramid geometry (τ_5_ is equal 0.27 for **2** and 0.26 for **3** [45]). The oxygen atoms of the *trans*-coordinated L^−^ constitute the base plane of the coordination polyhedrons and the Zn(II) atom strays from the equatorial plane by 0.38 and 0.39 Å for **2** and **3**, respectively. The axial positions are occupied by the oxygen atom of the water molecule. It is noteworthy that the bond length of the C-O from acyl fragments (1.255–1.262 Å) are noticeably shorter than the similar bond in the pyrazole fragments (1.273–1.283 Å), which is consistent with the deprotonation of the ligand in the enol form [46,47].

The crystal packing of the molecules is determined by noncovalent interactions, including H-bonds between the coordinated water molecule and the two N atoms of pyrazol moieties of two adjacent complex molecules, stacking interactions of the pyrazol rings, as well as intra- and intermolecular C-H…O/N and intermolecular C-H…π contacts (Tables S3–S5). In both cases of crystal packing, periodic two-dimensional lipid-like layers are formed due to oppositely directed alkyl fragments (Figure 2c,d).

The thin films were obtained by different ways: by thermal evaporation (complexes **1** and **2**), by spin coating (complexes **1**, **2** and **3**) and by Langmuir-Blodgett techniques (complex **3**). The morphology and the surface roughness of the thin films were used to examine by Atomic force microscopy (AFM) techniques. In order to study the chemical and phase composition of the films, EDX, PXRD analyses and infrared (IR) spectroscopy were conducted.

**Figure 3:**
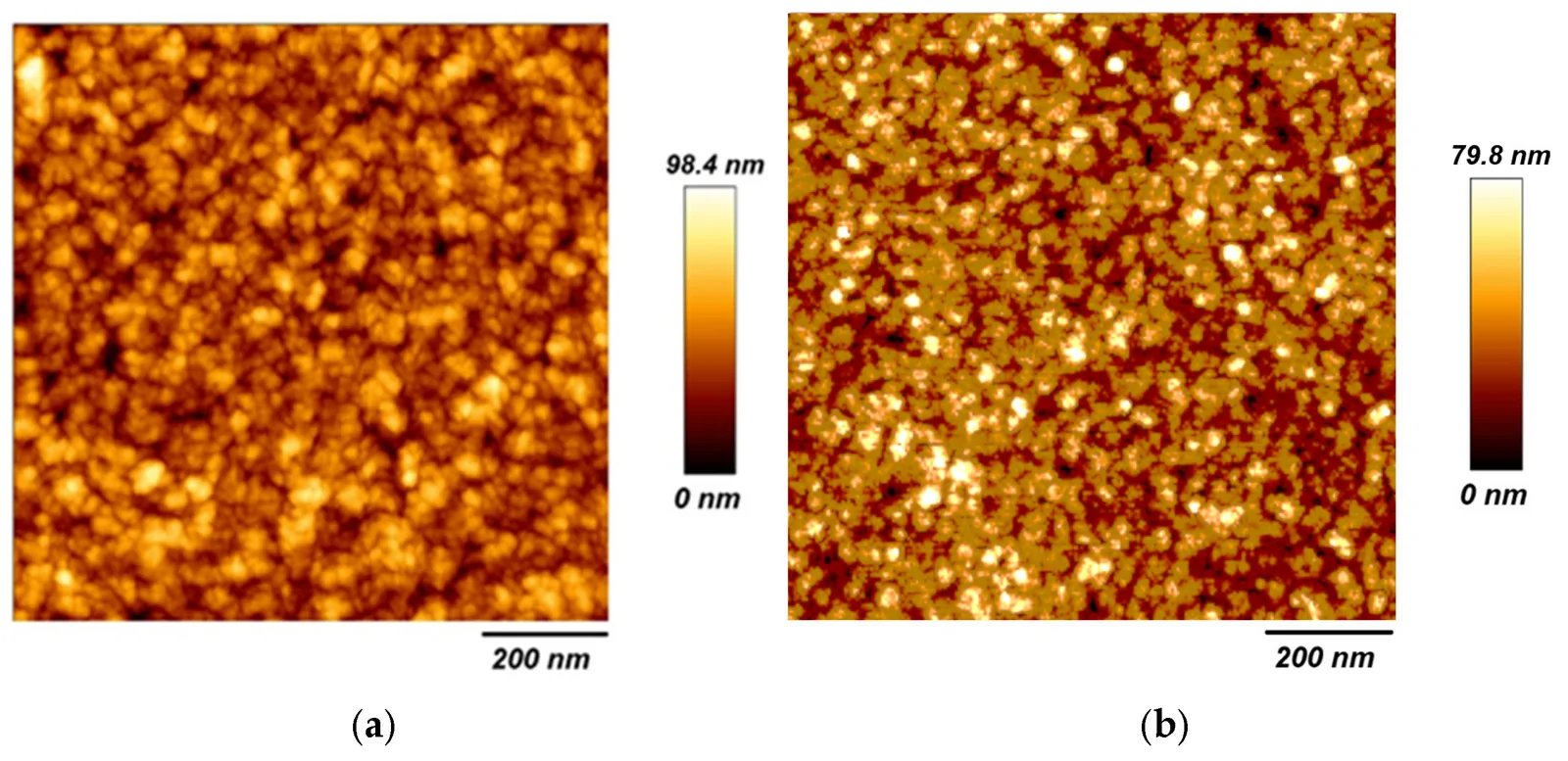
AFM images of thin films **1** (**a**) and **2** (**b**) obtained by TVD.

Experiments involving vacuum sublimation in a furnace with a gradient tube (T = 160 °C) showed that compounds **1** and **2** partially sublimed, resulting in only one condensation zone. For complex **3**, sublimation was not observed, presumably due to its high molar mass. The AFM images of the thin films of **1** (220 nm thick) and **2** (180 nm thick) deposited on glass substrate are shown in Figure 3. The images reveal that the thin films produced by thermal vacuum deposition (TVD) possess a noticeably heterogeneous surface. The films exhibit nano-spherical granules with grain sizes ranging from 20 to 30 nm. The root-mean-square roughness (*R*_q_) (20.4 nm for **1** and 24.7 nm for **2**) are relatively high, suggesting unevenness with the surface roughness.

**Figure 4:**
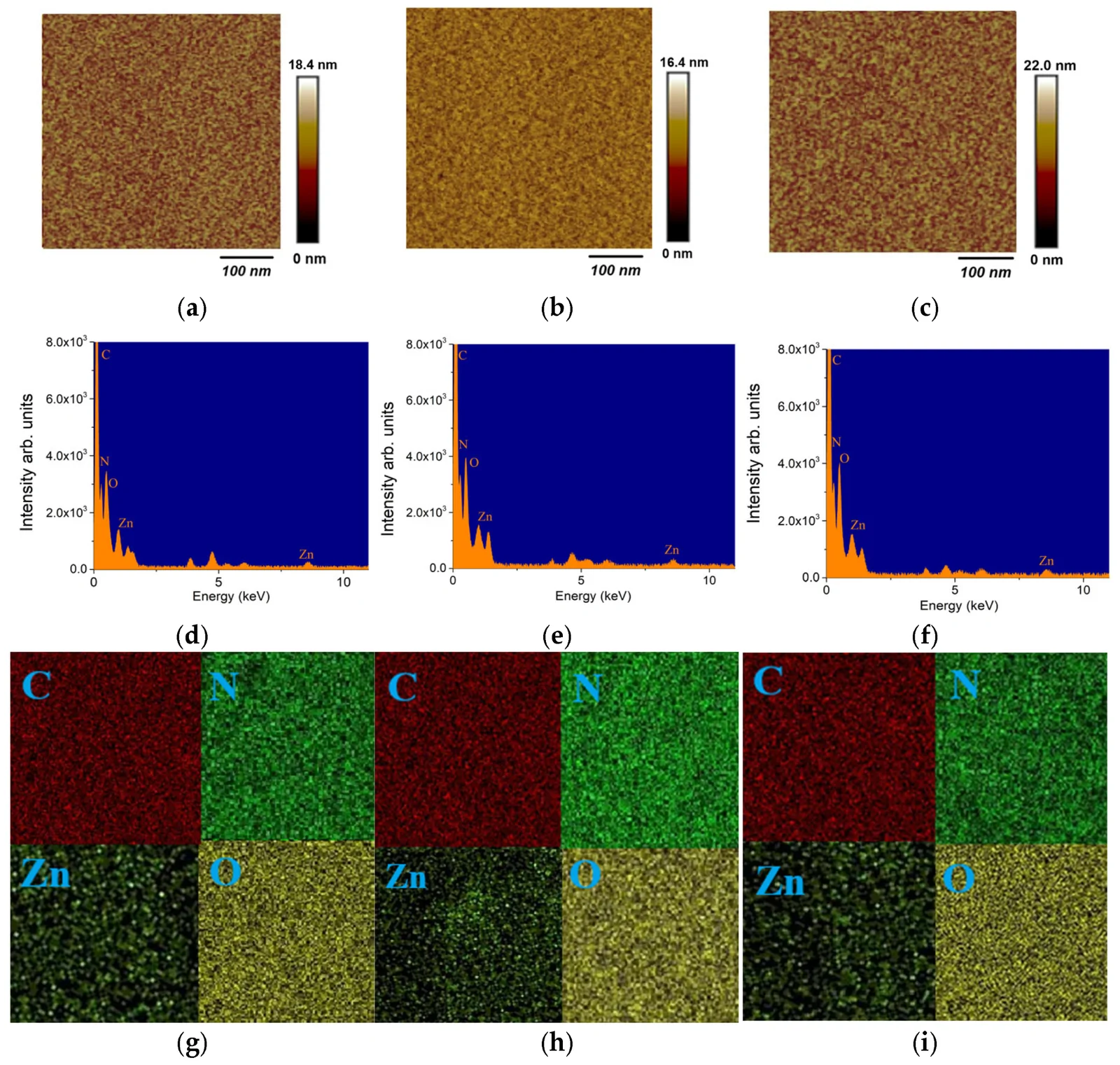
AFM images of thin films 1 (**a**), 2 (**b**) and 3 (**c**) obtained by TVD; EDX profile of thin films 1 (**d**), 2 (**e**) and 3 (**f**); EDX element maps of thin films 1 (**g**), 2 (**h**) and 3 (**i**).

When obtaining thin films by spin-coating, the spin speed, concentration, and nature of the solvent were varied. Given the low solubility of complexes in low-polar solvents, we used acetonitrile, 1,2-dichloroethane, and methanol as solvents to obtain high-quality thin films. The best results were achieved using 1,2-dichloroethane. AFM surface analysis of the thin films produced by spin-coating shows a smoother surface and greater homogeneity and density compared to those previously obtained via the TVD method (Figure 4). The average roughness remains relatively low (*R*_q_ ≈ 1.1–1.8 nm), suggesting a smooth and uniform surface morphology.

**Figure 5:**
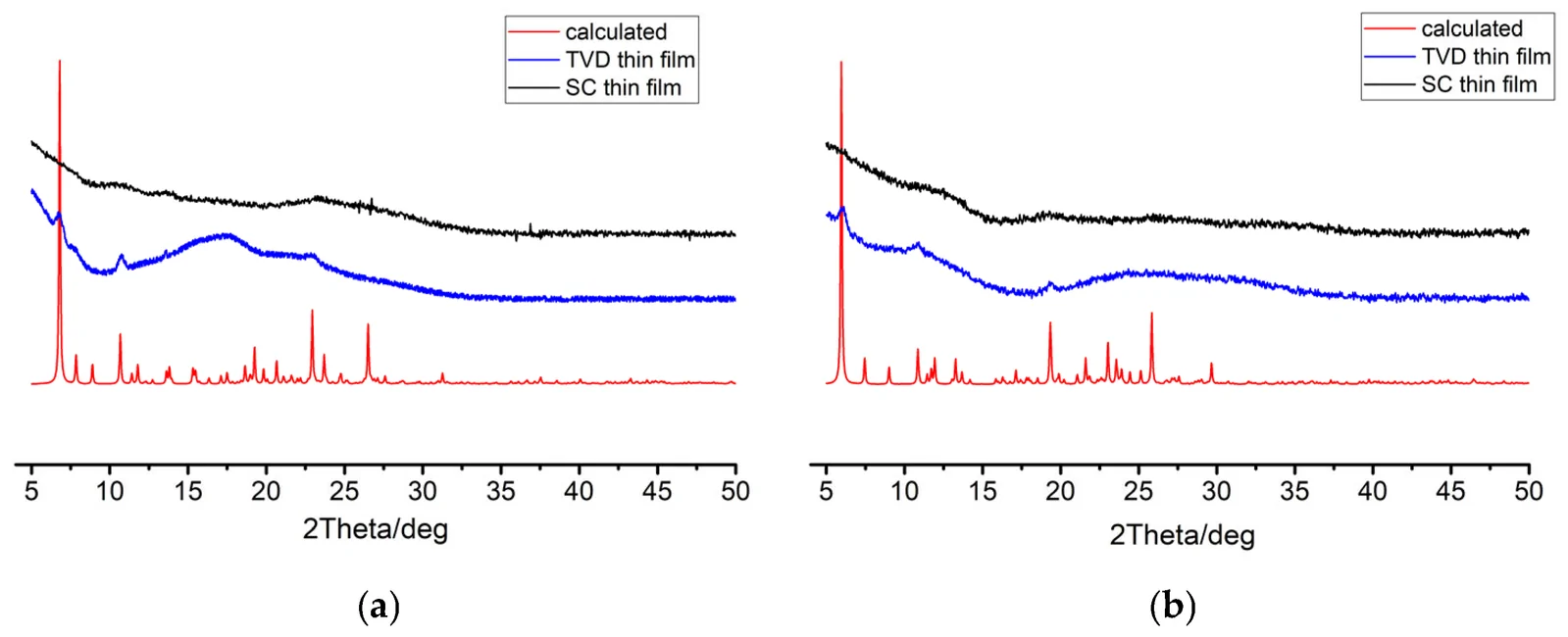
XRD patterns of the vacuum deposit and spin coated thin films of **1** (**a**) and **2** (**b**).

The most significant differences between the films obtained by the TVD and SCM methods are in the phase composition, as established by powder X-ray diffraction. The X-ray diffraction patterns of the thin films of complexes **1** and **2**, prepared via vacuum deposition and spin-coating, are shown in Figure 5. The presented patterns indicate that the structure of the thin films of complexes **1** and **2**, produced by the spin-coating method, predominantly exhibits an amorphous nature. In contrast, the XRD patterns of films obtained by vacuum deposition display narrow peaks, indicative of the formation of a polycrystalline phase. It should be noted that the vacuum deposition method generally produces films of higher quality compared to the spin-coating method. However, a comparison of the film quality for complexes **1** and **2** obtained by different methods shows that, in this case, spin-coating resulted films with lower surface roughness. Considering the XRD data indicating a high degree of crystallinity of the films produced by TVD method—it can be hypothesized that during film formation, the rate of nucleation centers formation lags behind the crystal growth rate. This leads to the formation of discrete structures on the substrate surface. The uniformity of TVD films could be improved by more careful optimization of the deposition parameters or post-synthetic treatment via annealing, which will be the subject of further studies.

EDX analysis showed the presence of C, N, O, and Zn in the layer and is consistent with the composition of the complexes that indicates the suitability of the spin-coating technique for preparing stable thin films of the complexes **1**–**3**. Moreover, characterization of materials by IR spectra, indicates the presence of the characteristic peaks for the ligands at 2950–2840 cm^−1^ from stretching frequencies of the C-H bond of aliphatic groups the positions of which correspond to the positions in the bulk samples. The above-described data indicates the suitability of the TVD and SCM techniques for the creation of thin films with the preservation of the molecular structure of complexes **1**–**3**.

**Figure 6:**
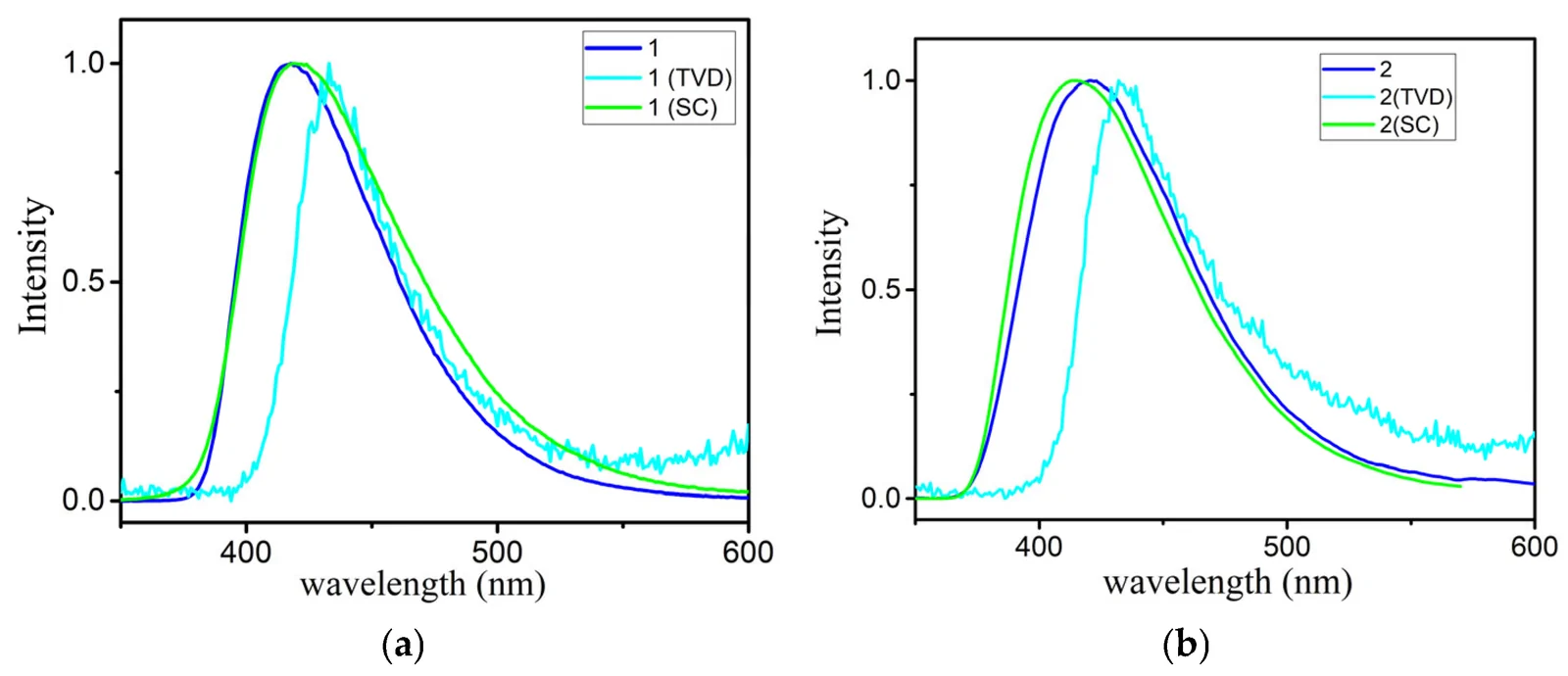
Luminescence spectra of the vacuum deposit and spin coated thin films of **1** (**a**) and **2** (**b**).

The fluorescence properties of the complexes **1** and **2** for the films obtained in different ways were also studied (Figure 6). Upon excitation at a wavelength of 330 nm, amorphous thin films prepared by spin-coating exhibit photoluminescence with maxima at 419 nm and 413 nm, values close to those observed in solutions. The quantum yield is 8.6% and 10.9%, respectively. Notably, polycrystalline films, obtained via vacuum deposition, display a bathochromic shift compared to the amorphous films, with emission maxima at 433 nm and 432 nm, and have relatively low quantum yields of 0.8% and 0.9% for samples **1** and **2**, respectively. Therefore, an influence of molecular packing in the solid phase on the optical properties can be concluded.

**Figure 7:**
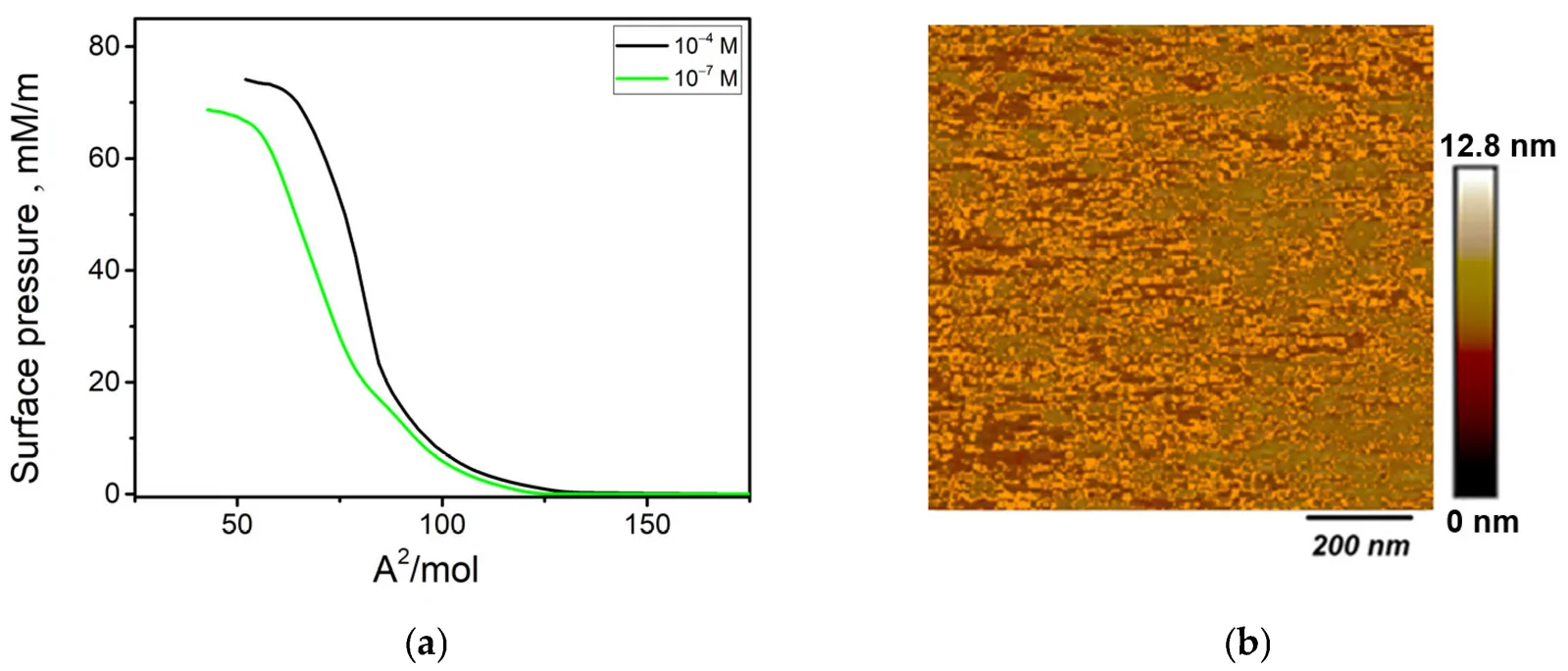
(**a**) Surface pressure-area (π-A) isotherms for **3** at different concentration; (**b**) AFM image of thin film **3** obtained by LBT.

The presence of a nonpolar alkyl “tail” and a polar coordination polyhedron allows for the production of thin films using the Langmuir–Blodgett technique [48,49,50]. When spread on the water surface, complexes **1** and **2** do not form monomolecular monolayers, and remain as separate aggregates at the water–air interface. In general, this result is consistent with the relatively short aliphatic chains, which are insufficient in length to give the complex an amphiphilic character. The surface pressure change as a function of surface area (Q-A) isotherm of complex **3** monolayer at the air–water interface at room temperature is shown in Figure 7a. It was found to be reproducible and exhibits the three characteristic regions corresponding to gaseous, liquid-expanded and liquid-condensed phases.

The monolayers of complex **3** were characterized by good compressibility and a well-defined crystalline phase, which was evident from their compression isotherms (Figure 7a). However, the minimum area per molecule in the condensed state varied depending on the concentration of the subphase complex. A_o_ values defined for **3** on subphases increase from 88.4 Å^2^/mol at a concentration of 10^−7^ M to 97.6 Å^2^/mol at 10^−5^ M and then remain constant up to a concentration of 10^−3^ M. The monomolecular single layer film was transferred to a glass substrate previously immersed in an aqueous sub-phase at a lifting speed of 1 mm/min. The monolayer was dried for 10 min in air and then a second layer was applied by dipping the single layer film at a rate of 1 mm/min. The process was repeated 8 times to obtain a 16-layer film. AFM surface analysis shows the presence of defects, which is indicative of an inefficient transfer of the film onto the substrate in the final stages. The AFM image clearly shows the formation of a non-uniform surface with roughness parameters in the range Rq = 1.87–8.40 nm. Heterogeneity is probable due to inefficient transfer of the film onto the substrate in the final stages. This fact indicates that for the creation of thin films based on complex **3**, the LB technique is inferior to the spin-coating method in terms of film quality and ease of creation.

## 3. Materials and Methods

All starting reagents and chemicals of analytical grade were purchased from either Aldrich (München, Germany) or Merck (Darmstadt, Germany) and used without further purification. Solvents used for spectroscopic studies were purified and dried according to standard procedures before use. 1-phenyl-3-methyl-4-acyl-5-pyrazolones (HL^1^-HL^3^) were prepared according to the literature method [51].

Elemental analyses of C, H, and N were performed with the EuroEA 3000 analyzer (EuroVector S.p.A., Cinisello Balsamo, Italy). The IR spectra were measured by the FSM 2202 spectrometer (OKB SPECTR LLC, Saint Petersburg, Russia) in the range of 4000–400 cm^−1^. UV–Vis spectra were recorded with a Cintra-3000 spectrophotometer (GBC Scientific Equipment, Melbourne, Australia) for the solid-state samples. Photoluminescence and excitation spectra were recorded on the FluoroMax-4 spectrofluorometer (Horiba Scientific, Edison, NJ, USA). The thermal behavior of the compound was studied using the simultaneous thermal analysis (STA) technique for parallel recording of TG (thermogravimetry) and DSC (differential scanning calorimetry) curves. RXPD data at room temperature were collected using a SuperMini 200, Rigaku (CuKα, λ = 1.54 Å) (Tokyo, Japan).

The flat glass slides used as substrates were pre-cleaned by chromic acid for 10 h and then washed several times using distilled water. After that, they were immersed in isopropyl alcohol and washed in distilled water and acetone and finally dried in dry air.

Thermal vacuum deposition of 1 and 2 was performed on a BOC Edwards Auto 306 equipment. The deposition process was carried out in a vacuum chamber by heating complexes **1** and **2** in a molybdenum tube at 150 °C and condensing the vapors onto a pre-cleaned glass surface at a residual pressure of ~10^−5^ mbar and with deposition rates of 0.5 nm/s.

Spin coating deposition was performed at 2500 rpm for filtered solution of 1 mg complexes **1**–**3** in 50 mL of 1,2-dichloroethane. After deposition, thin films were dried under a nitrogen atmosphere at 50 °C during 30 min in order to remove the residual amounts of the solvent and stabilize the films.

π–A compression isotherms and Langmuir–Blodgett films (LBF) transfer were performed at a temperature of 20 °C on a KSV Minitrough II (KSV Instruments, Helsinki, Finland) equipped with a platinum Wilhelmy plate. Compression isotherms of **3** were recorded on aqueous subphases containing 1 × 10^−4^ of the complex. LBFs were formed by the vertical transfer of monolayers from the surface of the aqueous subphase onto glass plates. A 16-layer ZnL^3^_2_ LBF was obtained. The orientation of the layers in the obtained LBFs corresponded to the Y-type.

A quartz detector SQM 160 (INFICON, Bad Ragaz, Switzerland) controlled the evaporation speed and thickness of the deposited layers. Atomic force microscopy (AFM) was used to examine the topographical properties of the thin films. The AFM imaging was performed at room temperature in contact mode by using Nanosurf easy Scan 2 AFM with a SICON-A cantilever microscope (Liestal, Switzerland) in the hard tapping mode. Elemental analyses of the as-deposited thin film were performed using an energy-dispersive X-ray (EDX) microanalysis unit of a field emission scanning electron microscope FE-SEM (QUANTA FEG250, FEI Company, Eindhoven, The Netherlands) at an operating voltage of 20.00 kV.

### 3.1. Synthetic Procedures

The related ligands (HL^1^-HL^3^) 2 mmol and Zn(CH_3_COO)_2_ 2H_2_O (219 mg, 1 mmol) were dissolved in 40 mL CH_3_OH, and stirred at 60 °C. After 1 h, white precipitate was formed and crude compounds were collected by filtration. Pure crystalline samples as well as single crystals for XRD were obtained by recrystallization of crude products from MeOH.

[Zn(L^1^)_2_(H_2_O)] (**1**) Yield 72%. Anal. calc. (%) for C_34_H_44_N_4_O_5_Zn: C, 62,43%; H, 6,78%; N, 8,56%. Found (%): C, 62.62; H, 6.49; N, 8.74. IR (cm^−1^): 3347w, 2955s, 2919s, 2852s, 1624s, 1594 m, 1498s, 1441m, 1357m, 1072m, 753m, 510w.

[Zn(L^2^)_2_(H_2_O)] (**2**) Yield 73%. Anal. calc. (%) for C_40_H_56_N_4_O_5_Zn: C, 65.07; H, 7.64; N, 7.59. Found (%): C, 64.57; H, 7.50; N, 7.81. IR (cm^−1^): 2918s, 2850s, 1611s, 1595 m, 1502s, 1467m 1441m, 1371m, 1079m, 1069m, 752m, 509w.

[Zn(L^3^)_2_(H_2_O)] (**3**) Yield 68%. Anal. calc. (%) for C_52_H_80_N_4_O_5_Zn: C, 68.89; H, 8.89; N, 6.28. Found (%): C, 68.74; H, 8.61; N, 6.18. IR (cm^−1^): 2955w, 2919s, 2850s, 1612s, 1595 m, 1503s, 1463m, 1373m, 1079m, 753m, 510w.

The single crystal X–ray diffraction data for **2** and **3** were collected using the Bruker D8 Venture diffractometer equipped with a CCD detector and a micro-focus MoKα radiation source (λ = 0.71073 Å). Semi-empirical absorption correction was applied for both samples [52]. The structure was solved by direct methods and refined in the full-matrix anisotropic approximation for all non-hydrogen atoms. The structure **2** was solved taking int account the disorder of the terminal C_3_H_7_ and C_4_H_9_ fragments of coordinated ligands with an occupancy ratio of 678(5):0.322(5). The hydrogen atoms of the carbon-containing ligand were positioned geometrically and refined by using a riding model. All the calculations were performed by direct methods and using the SHELX-2018 and OLEX-2 program package [53,54]. The crystallographic parameters and the structure refinement statistics are shown in Table S1. CCDC numbers 2563712 for **2** and 2563713 for **3** contain the supplementary crystallographic data for the reported compounds. These data can be obtained free of charge from The Cambridge Crystallographic Data Centre via http://www.ccdc.cam.ac.uk/data_request/cif (accessed on 15 May 2026).

### 3.2. Computational Details

The simulation of the UV absorption spectra for the complexes was performed using time-dependent density functional theory (TD-DFT). The calculations accounted for solvent effects employing the polarizable continuum model (PCM) [55]. All calculations were carried out using the GAUSSIAN-09 program package [56]. Fifty excited states were calculated with the Becke’s two-parameter hybrid exchange functional [57] combined with the Perdew correlation functional (BP86) [58] and the standard split-valence polarized 6-31G(d) basis set [59,60].

## 4. Conclusions

In summary, we successfully synthesized coordination compounds of zinc with three acylpyrazolones. The complexes were thoroughly characterized using IR and UV–Vis spectroscopy, thermogravimetric analysis (TGA-DSC) and luminescence spectroscopy. It was demonstrated that the complexes are weak emitters in the polycrystalline state and exhibit satisfactory luminescence efficiency in solutions and thin films. The X-ray structures for the compounds were determined. The synthesized complexes were used to create thin films based on them using thermal vacuum deposition, spin-coating, and Langmuir–Blodgett techniques. It was found that for complexes with relatively short aliphatic substituents (R—C_6_H_13_ and C_9_H_19_), the preferred methods are vacuum deposition and spin-coating. Films produced by TVD (thermal vacuum deposition) exhibited a high degree of crystallinity, whereas spin-coated films were uniform and amorphous. When the length of the aliphatic substituent in the acyl fragment increased to R—C_15_H_31_, the most suitable film-forming methods were spin-coating and Langmuir–Blodgett.

## Data Availability

The original contributions presented in this study are included in the article/Supplementary Materials. Further inquiries can be directed to the corresponding author.

## Supplementary Materials

The following supporting information can be downloaded at: https://www.mdpi.com/article/10.3390/ijms27177560/s1. Reference [61] is cited in Supplementary Materials.
