# Supplementary material for: Zn Complexes with the 4-Acyl-Pyrazol-5-One Basis and Their Thin Films
Source: Int J Mol Sci. 2026 Aug 24;27(17):7560. doi: 10.3390/ijms27177560 (PMC13566104; doi:10.3390/ijms27177560)
Supplement: Supplementary file 1 [file ijms-27-07560-s001.zip › ijms-4423048-supplementary.pdf]

Supplementary Materials to the paper entitled:

Zn complexes with the 4-acyl-pyrazol-5-one basis and their thin films

Alexey Gusev, Elena Braga, Alexandra Pismennaya, Valery Vlasenko, Miki Hasegava, Mikhail Kiskin, Wolfgang Linert

Table S1. Crystal data and structure refinements for **2** and **3**

Parameters	**2**	**3**
Crystal data		
Chemical formula	$C_{40}H_{56}N_4O_5Zn$	$C_{52}H_{80}N_4O_5Zn$
M_r	738.25	906.57
Crystal system, space group	Triclinic, $P\bar{1}$	Triclinic, $P\bar{1}$
Temperature (K)	100	100
a, *b*, *c* (Å)	10.0407(3), 12.5895(4), 16.0084(5)	10.3085 (19), 12.460 (2), 19.609 (4)
α, β, γ (°)	70.3670(10), 77.6790(10), 83.595(10)	84.437 (6), 81.890 (6), 82.038 (6)
V (Å^3)	1860.29(10)	2461.8 (8)
Z	2	2
μ (mm^{-1})	0.71	0.55
ρ (g/cm^3)	1.318	1.223
Crystal size (mm)	0.34 × 0.20 × 0.10	0.20 × 0.04 × 0.02
T_{min}/T_{max}	0.6760/0.7466	0.5998/0.7465
No. of measured, independent and observed [$I > 2\sigma(I)$] reflections	22412, 12511, 9475	18837, 9626, 6154
R_{int}	0.028	0.056
$(\sin \theta/\lambda)_{max}$ (Å^{-1})	0.772	0.617
$R[F^2 > 2\sigma(F^2)]$, $wR(F^2)$, S	0.059, 0.153, 1.12	0.051, 0.110, 0.94
No. of parameters	524	566
No. of restraints	61	1
$\Delta\rho_{max}$, $\Delta\rho_{min}$ (e Å^{-3})	1.22, -1.26	0.34, -0.55

Table S2. Selected geometric parameters for **2** and **3**

2	3
Bond's Length (Å)	
Zn1-O1 1.9764(19)<br>Zn1-O2 2.094(2)<br>Zn1-O3 1.9639(18)<br>Zn1-O4 2.0674(19)<br>Zn1-O1W 1.9809(19)	Zn1-O1 1.9764(19)<br>Zn1-O2 2.094(2)<br>Zn1-O3 1.9639(18)<br>Zn1-O4 2.0674(19)<br>Zn1-O1W 1.9809(19)
Valence angles	
O1-Zn1-O2 88.53(8)<br>O1-Zn1-O3 149.52(8)<br>O1-Zn1-O4 83.09(8)<br>O1-Zn1-O1W 108.88(8)<br>O2-Zn1-O3 90.87(7)<br>O2-Zn1-O4 165.38(8)<br>O2-Zn1-O1W- 95.50(8)<br>O3-Zn1-O4 90.33(7)<br>O3-Zn1-O1W 101.52(8)<br>O4-Zn1-O1W 98.54(9)	O1-Zn1-O2 88.53(8)<br>O1-Zn1-O3 149.52(8)<br>O1-Zn1-O4 83.09(8)<br>O1-Zn1-O1W 108.88(8)<br>O2-Zn1-O4 90.87(7)<br>O2-Zn1-O3 165.38(8)<br>O2-Zn1-O1W 95.50(8)<br>O3-Zn1-O4 90.33(7)<br>O3-Zn1-O1W 101.52(8)<br>O4-Zn1-O1W 98.54(9)

Table S3. Parameters of D-H···A interactions in **2** and **3** (the analysis was done using PLATON software [A.L.Spek, Acta Cryst. 2009, D65, 148-155]).

Interaction	D-H, Å	H…A, Å	D…A, Å	D-H-A, deg.	Interaction
**2**					
O5-H5A...N2	2-x,1-y, 1-z	0.85	1.94	2.735(3)	155
O5-H5B...N4	1-x, 2-y, 1-z	0.85	1.94	2.764(3)	162
C5-H5...O1	-	0.95	2.19	2.844(3)	125
C9-H9...N2	-	0.95	2.45	2.798(3)	101
C12-H12A...O3	1-x, 1-y, 1-z	0.99	2.56	3.524(3)	165
C24-H24...O3	-	0.95	2.18	2.825(3)	124
C28-H28...N4		0.95	2.48	2.814(3)	101
**3**					
O1W-H1WA...N4	2-x, 1-y, 1-z	0.87	1.88	2.725(3)	162
O1W-H1WB...N2	1-x, 2-y, 1-z	0.87	1.90	2.736(3)	162
C5-H5...O1	-	0.95	2.21	2.849(4)	124
C9-H9...N2	-	0.95	2.48	2.813(4)	101
C31-H31...O3	-	0.95	2.22	2.847(4)	123
C35-H35...N4	-	0.95	2.48	2.812(4)	100
C38-H38B...O1	1-x, 1-y, 1-z	0.99	2.59	3.507(3)	155
C41-H41A...O3	1-x, 1-y, 1-z	0.99	2.55	3.351(4)	138

Table S4. Selected parameters of π…π intermolecular interactions in **2** and **3** ($C_g…C_g$ = distance between ring centroids (Å); α = dihedral angle between planes I and J (deg.); C_g(I)_Perp = Perpendicular distance of Cg(I) on ring J (Å); Slippage = distance between Cg(I) and perpendicular projection of Cg(J) on ring I (Ang) (Å); the analysis was done using PLATON software [A.L.Spek, Acta Cryst. 2009, D65, 148-155]).

Interaction	Symmetry	$C_g…C_g$, Å	α, deg.	C_g(I)_Perp, Å	Slippage, Å
		**2**			
Cg_1(N1N2C2C3C1)…Cg_1	2-x, 1-y, 1-z	3.4127(13)	0.00(12)	3.3962(9)	0.335
Cg_2(N3N4C21C22C20)…Cg_2	1-x, 2-y, 1-z	3.6044(13)	0.03(12)	3.5597(9)	0.566
		**3**			
Cg_1(N1N2C2C3C1)…Cg_1	1-x, 2-y, 1-z	3.6559(18)	0.02(16)	3.6312(12)	0.424
Cg_2(N3N4C28C29C27)…Cg_2	2-x, 1-y, 1-z	3.5490(18)	0.00(16)	3.4758(12)	0.717

Table S5. Parameters of C–H… π interactions in **2** and **3** (Cg is centroid of 5,6-memmberd ring; H-Perp is perpendicular distance of H on ring; γ is angle H->Cg vector and normal to ring plane; the analysis was done using PLATON software [A.L.Spek, Acta Cryst. 2009, D65, 148-155]).

Interaction	Symmetry	H...Cg, Å	γ, deg.	C-H…Cg, deg.	C…Cg, Å
		**2**			
C12-H12B…Cg_6(C4-C9)	2-x, 1-y, 1-z	2.90	1.00	140	3.712(2)
C30-H30B…Cg_5(C23-C28)	1-x, 2-y, 1-z	2.70	8.34	144	3.545(2)
C38-H38B…Cg_2(N3N4C21C22C20)	1-x, 1-y, 2-z	2.90	15.41	164	3.859(5)
C40A-H40C…Cg_2(N3N4C21C22C20)	1-x, 1-y, 2-z	2.94	9.51	140	3.749(9)
		**3**			
C12-H12A…Cg5(C4-C9)	1-x, 2-y, 1-z	2.83	8.58	141	3.657(3)
C38-H38A…Cg6(C30-C35)	2-x, 1-y, 1-z	2.94	13.11	141	3.768(3)

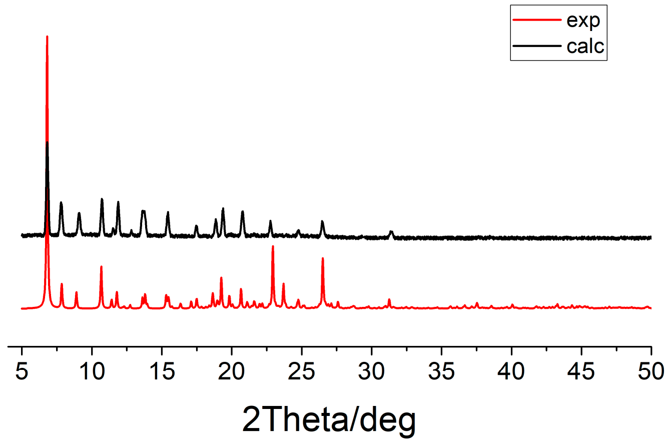

Figure S1. PXRD data of 1

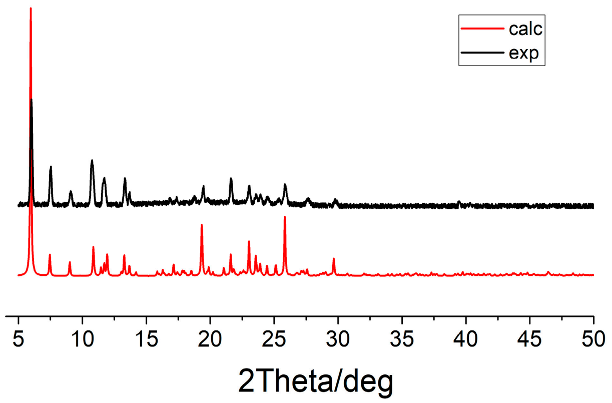

Figure S2. PXRD data of 2

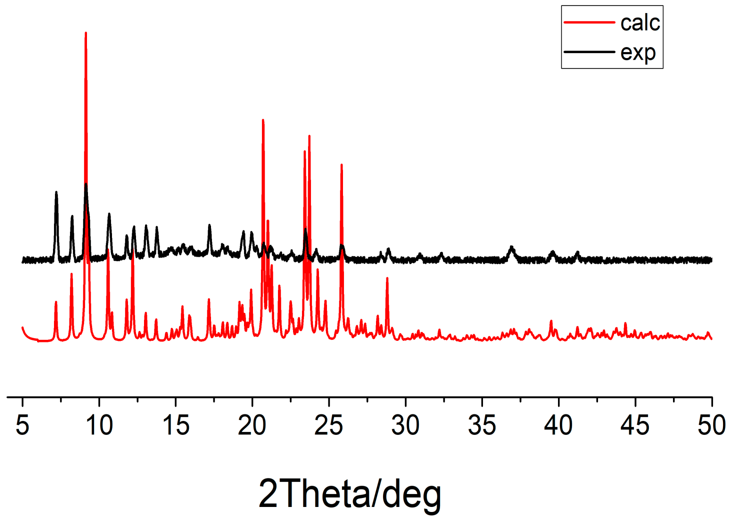

Figure S3. PXRD data of 3

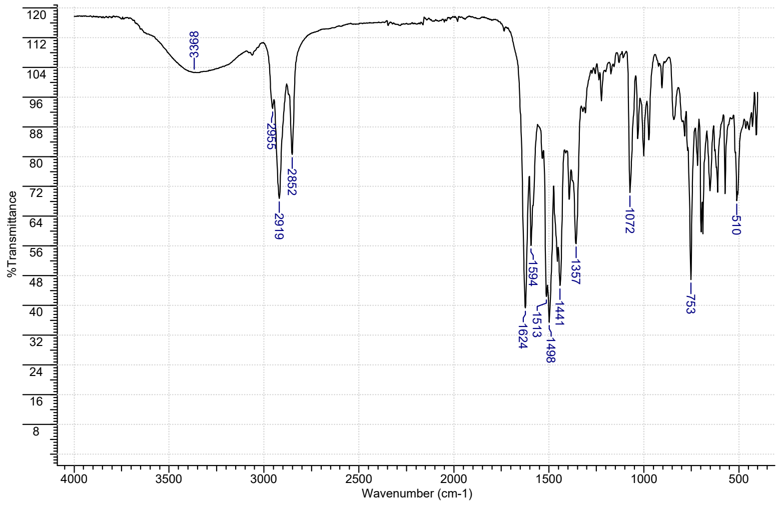

Figure S4. IR spectra of 1

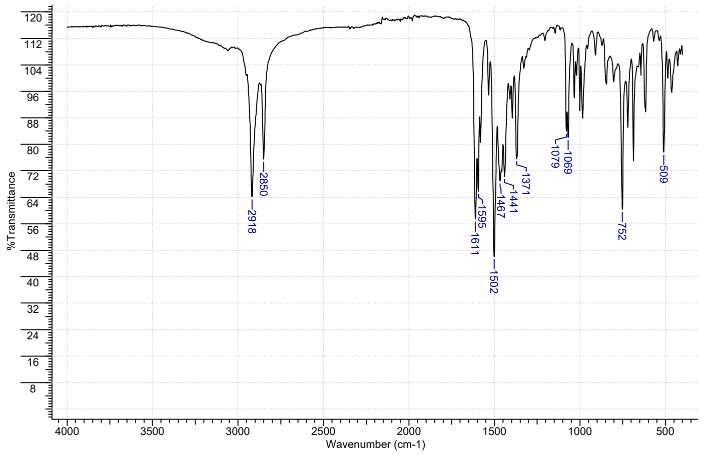

Figure S5. IR spectra of 2

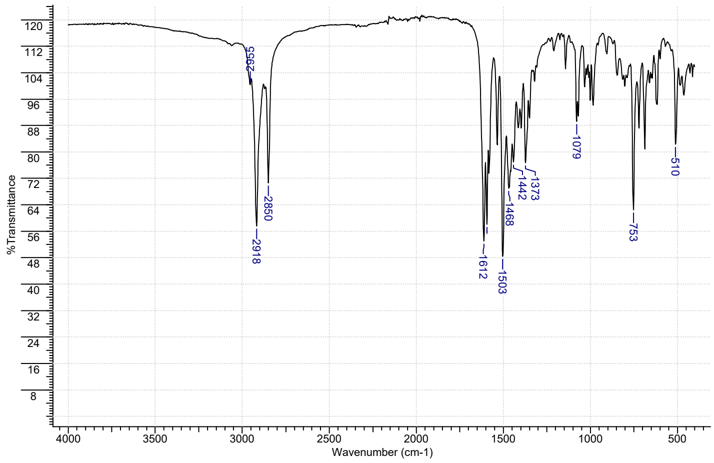

Figure S6. IR spectra of 3

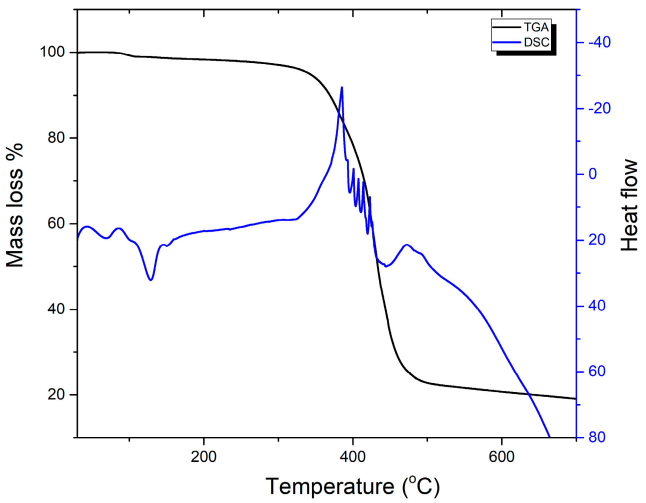

Figure S7. TGA and DSC curves of 1

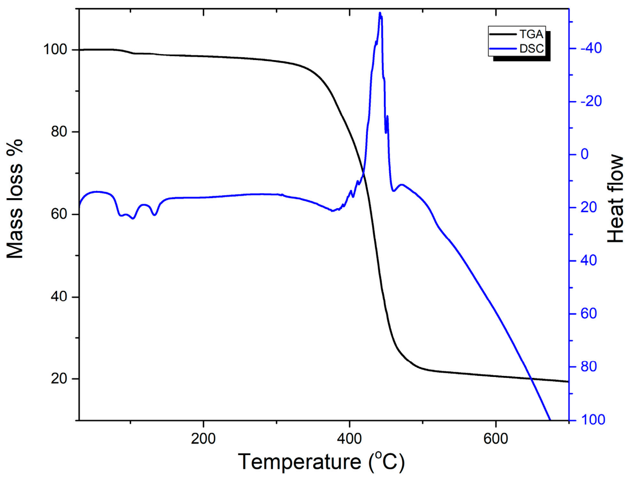

Figure S8. TGA and DSC curves of 2

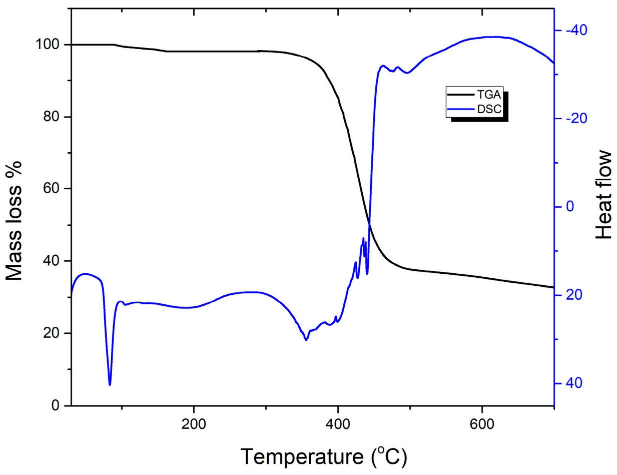

Figure S9. TGA and DSC curves of 3
